# Cadmium Sulfide Nanoparticles: Preparation, Characterization, and Biomedical Applications

**DOI:** 10.3390/molecules28093857

**Published:** 2023-05-02

**Authors:** Alireza Ghasempour, Hamideh Dehghan, Mehrnaz Ataee, Bozhi Chen, Zeqiang Zhao, Mahsa Sedighi, Xindong Guo, Mohammad-Ali Shahbazi

**Affiliations:** 1 Student Research Committee, Birjand University of Medical Sciences, Birjand 9717853076, Iran; 2 Beijing Laboratory of Biomedical Materials, College of Materials Science and Engineering, Beijing University of Chemical Technology, Beijing 100029, China; 3 Department of Pharmaceutics and Nanotechnology, School of Pharmacy, Birjand University of Medical Sciences, Birjand 9717853076, Iran; 4 Cellular and Molecular Research Center, Birjand University of Medical Sciences, Birjand 9717853076, Iran; 5 Department of Biomedical Engineering, University Medical Center Groningen, University of Groningen, Antonius Deusinglaan 1, 9713 AV Groningen, The Netherlands; 6 W.J. Kolff Institute for Biomedical Engineering and Materials Science, University of Groningen, Antonius Deusinglaan 1, 9713 AV Groningen, The Netherlands

**Keywords:** cadmium sulfide nanoparticles, nanobiotechnology, preparation methods, characterization, biomedical applications

## Abstract

Cadmium sulfide nanoparticles (CdS NPs) have been employed in various fields of nanobiotechnology due to their proven biomedical properties. They are unique in their properties due to their size and shape, and they are popular in the area of biosensors, bioimaging, and antibacterial and anticancer applications. Most CdS NPs are generally synthesized through chemical, physical, or biological methods. Among these methods, biogenic synthesis has attracted more attention due to its high efficiency, environmental friendliness, and biocompatibility features. The green approach was found to be superior to other methods in terms of maintaining the structural characteristics needed for optimal biomedical applications. The size and coating components of CdS NPs play a crucial role in their biomedical activities, such as anticancer, antibacterial, bioimaging, and biosensing applications. CdS NPs have gained significant interest in bioimaging due to their desirable properties, including good dispersion, cell integrity preservation, and efficient light scattering. Despite these, further studies are necessary, particularly in vivo studies to reduce NPs’ toxicity. This review discusses the different methods of synthesis, how CdS NPs are characterized, and their applications in the biomedical field.

## 1. Introduction

Nanotechnology is cutting-edge technology and has become one of the emerging multidisciplinary fields receiving universal attention and playing a considerable role in medicine and pharmacology [1]. There is a great deal of interest and privileged status given to nanoparticles (NPs) among nanostructures today [2]. The broad category of materials known as NPs includes particulate compounds that have at least one dimension in size ranging from 1 to 100 nm. Scientists discovered that size could affect the physicochemical characteristics of substances, which led them to recognize the significance of these materials [3]. They found out that, compared to larger sizes of the relevant materials, the qualities of the NPs, which are commonly categorized as organic, inorganic, and carbon-based particles, are enhanced [4,5]. For instance, their high surface-to-volume ratio causes an exponential rise in the reactivity at the molecular scale [5,6,7]. NPs are proving to be effective means with a wide range of applications in the biological and non-biological fields of drug delivery, diagnostics, cosmetics, agriculture, and other areas of science [7,8,9,10,11].

Metallic NPs are available in various types, including gold, silver, alloys, magnetic, etc. [12,13]. Cadmium (Cd) has unique properties, such as high electrical conductivity, corrosion resistance, flexible, and high malleability [14,15]. No biological function has been found for Cd, and its toxicity to humans has been confirmed [16]. Reactive oxygen species (ROS) produced as a result of oxidative stress brought on by Cd bioaccumulation in human tissues can interfere with the antioxidant defense mechanism This, in turn, can lead to various health problems [17]. Moreover, cadmium sulfide (CdS) is a fluorescent material that is used in medicine due to its excellent optical and electrical properties, photocatalytic activity, and lower toxicity than Cd [18,19,20]. Chemical, physical, and biological methods create different types of CdS NPs. The application of CdS NPs is dependent on the characteristics of the synthesized NPs, such as size, shape, and surface charge, which are dependent on their synthesis method [15]. Because of their lower toxicity and excellent compatibility with biological systems, CdS NPs synthesized by biological methods are more commonly used in medical sciences [21]. The non-toxicity and the antioxidant, antimicrobial, anticancer, imaging probe, and drug delivery properties of CdS NPs have led to their use as drugs and diagnostic tools in vivo and in vitro models [22,23,24,25].

The present review highlights the synthesis and characterization strategies related to CdS NPs, followed by a comprehensive discussion on some specific focused biomedical applications of CdS NPs, including bioimaging, biosensors, anticancer, and antimicrobial activities.

## 2. Preparation Methods of Cadmium Sulfide Nanoparticles

The different types of CdS NPs are synthesized using various techniques, including chemical, physical, and biological methods [15]. Since new and unknown properties of nanomaterials depend on their size and shape, achieving new approaches for the fabrication of CdS NPs and identifying the mechanisms to control and modify the size and shape of the nanomaterials are fundamental challenges in nanochemistry. By controlling the thermodynamics and kinetics during the nucleation and growth of nanocrystals and modifying the parameters involved in various synthesis methods, high-quality NPs with suitable shapes, sizes, and other structural characteristics can be obtained [26]. Table 1 presents a comprehensive analysis of CdS NPs synthesized via four different methods, namely biogenic/green, chemical, physical, and physicochemical, and the important features of each method are highlighted. Furthermore, the table documents the characterization tests conducted in each study, demonstrating that CdS NPs have undergone a comprehensive analysis. According to Table 1, TEM, UV–Visible spectra, SEM, FTIR, and XRD are the most commonly used methods of characterization. Most NPs had a spherical shape, with sizes ranging from ~2–45 nm for biogenic, ~5–90 nm for chemical, and ~5–180 nm for physical NPs. Some structures larger than 100 nm were also reported.

Below, different methods to synthesize CdS NPs have been reviewed with several examples.

### 2.1. Biogenic Methods

Recently, the synthesis of NPs by the biogenic or green approach has been considered an alternative method to conventional methods. Biogenic approaches have received much attention due to their capacity to reduce the toxicity of NPs and their eco-friendly, cost-effective, easy, and fast process of synthesis. In addition, there is no need to use high temperatures, high energy and pressure, and the toxic chemicals that are usually used in chemical and physical methods [13,15]. For the biological synthesis of NPs, diverse sources such as plants and their metabolites (e.g., *Panicumsarmentosum* [42], *Dicliptera Roxburghiana* [41], *Asparagus racemosus* [79], etc.), as well as microorganisms such as algae (e.g., *Phormidium tenue* [80]), fungi (e.g., *Fusarium oxysporum f.* sp. *Lycopersici* [32], *Aspergillus niger* [36,39] and *Fusarium oxysporum* [39]), yeasts (e.g., *Trichosporon jirovecii* [29]) and bacteria (e.g., *Escherichia coli* [23,28,31,38,39], *Viridi bacillus arenosi* [30], *Rhodopseudomonas palustris* [33], *Bacillus licheniformis* [34,39,40], *Shewanella oneidensis* [35], *Pseudoalteromonas* [37], and *Klebsiella* sp. [38]) are utilized [81]. In general, the various compounds of biomaterials, such as proteins, alkaloids, flavonoids, polyphenols, etc., act as reducing and capping metal salt precursors [81,82].

**Figure 1:**
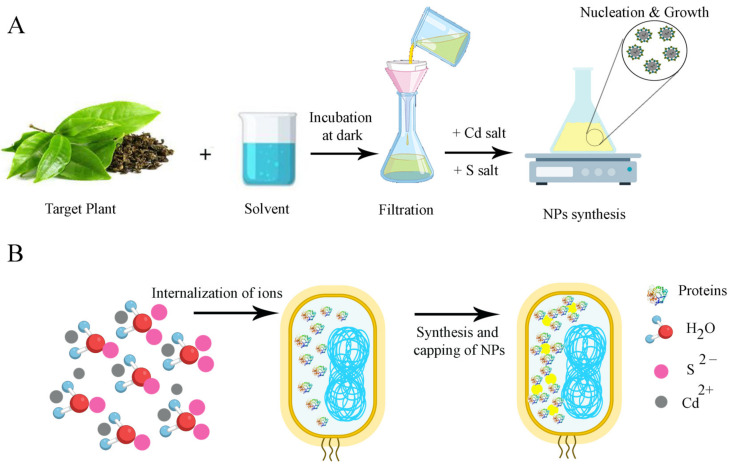
(**A**) schematic illustration of different stages in green synthesis of CdS NPs by plant extract. (**B**) Schematic illustration of the proposed mechanism for the biosynthesis of nanoparticles via bacteria.

To synthesize CdS NPs using plant extracts, the plant is selected based on its photochemical and biomedical properties [15]. After adding the prepared plant extract to the Cd salt, the biological reduction process takes place by phytochemicals already present in the extract, leading to the synthesis of CdS NPs. The obtained NPs are filtered and washed, and they are eventually dried for various uses (Figure 1A) [15,81].

Suranjit Prasad et al. used the leaf extract of *Asparagus racemosus* as a stabilizing and capping agent for the preparation of water-soluble CdS NPs. After the preparation of leaf extract, the green synthesis of CdS NPs was performed using a certain amount of sodium sulfide (2 mM) that was added dropwise into the solution of cadmium chloride and leaf extract, followed by being placed in a rotatory orbital shaker operating at 200 rpm, at 30 °C, for 12 h in dark condition. The formation of spherical, polydispersed, and crystalline CdS NPs with a diameter ranging from 2 to 8 nm was characterized using ultraviolet-visible (UV–Vis) spectrophotometry, transmission electron microscopy (TEM), etc. [79].

Shivaji et al. reported a green synthesis approach for fabricating CdS quantum dots (QDs) with a 2–5 nm particle size, using tea leaf extract as a toxic-free particle-stabilizing agent. To biologically synthesize CdS QDs, a certain amount of CdSO_4_ was added to the extract and incubated for three days in the dark condition, followed by adding Na_2_S and incubating for four days. The bright yellow color solution was then centrifuged and lyophilized for further characterization studies. Finally, their antibacterial activity was shown by a well-diffusion assay, and the cytotoxicity effect of CdS QDs was demonstrated against A549 cancer cells [43].

Microorganisms are also a significant source for the biomimetic synthesis of CdS NPs. After choosing a specific microorganism, the growth of the microbe is accomplished by producing a culture in an appropriate medium. Next, a solution including a combination of sodium sulfide and Cd salt is added to the culture. The Cd is biologically reduced by the microbes to produce NPs, which are isolated, washed with an appropriate solvent, and then characterized (Figure 1B) [15]. As an example, research was performed to assess the extracellular production of CdS QDs in *Fusarium oxysporum* f. sp. *Lycopersici*. To produce biologically synthesized CdS NPs (BS CdS NPs), the rinsed biomass of *F. oxysporum* f. sp. *Lycopersici* was put in 250 mL Erlenmeyer flasks with 62.5 mL of 1 mM CdSO_4_. At 0, 2, 3, 5, 9, and 12 days of incubation, samples of CdS NPs were collected. Filtration was used to separate the biomass for sampling, and then 0.5 mL of the filtrate was collected and stored at 4 °C for analysis. The rest of the filtrate and biomass were then combined to resume the incubation process at 30 °C with 150 rpm until the 12th day. A transmission electron microscope (TEM) study of biologically synthesized QDs revealed distinct, uniform-sized spherical NPs with diameters between 2 and 6 nm. The presence of S and Cd was verified by energy-dispersive spectroscopy [32].

As an additional example of microbial synthesis, Varmazyari et al. used the Gram-positive (G^+^) bacterium *Viridi bacillus arenosi* K64 to create CdS NPs. In this regard, 20 mL of CdCl_2_ and 5 mL of Na_2_SO_3_ were added to the bacterial suspension. For 10 to 20 min, the supernatant was warmed in a water bath at 60 °C until a yellow-white color developed as an indicator of successful synthesis [30].

### 2.2. Chemical Methods

#### 2.2.1. Chemical Precipitation Method

CdS NPs have been prepared in recent decades by using various wet chemical techniques, including chemical precipitation, solvothermal, micro-emulsion, and hydrothermal processes [38]. Due to its ease of synthesis, simplicity, short reaction time, easy processing, low-cost operation, and excellent purity, the chemical precipitation method is the most widely used methodology for producing CdS NPs [44,83].

In a study, the preparation of CdS NPs was performed at room temperature (RT), using a 0.1 M Na_2_S aqueous solution and CdSO_4_ in various molar concentrations of 0.1, 0.3, and 0.5 M. Each solution was independently dissolved in dH_2_O while being stirred for 10 min. The Na_2_S solution was gently added to the CdSO_4_ solution after complete dissolution, and the mixture was stirred for 20 min. A wet yellow precipitate was produced and saved throughout this technique [48].

Qutub et al. applied H_2_S, Na_2_S, and (NH_4_)_2_S as sulfide ion sources to create CdS NPs by using various chemical precursors. In an aqueous solution at standard pressure and temperature, CdS NPs were produced using typical chemical precipitation reactions. Six various synthesis reactions were carried out utilizing (NH_4_)_2_S, H_2_S, and Na_2_S as S^2-^ ion suppliers in the presence and absence of stabilizing factors to synthesize CdS NPs [44].

#### 2.2.2. Wet Chemical Synthesis

The wet chemical approach has been used extensively among the available methods of chemical synthesis, and it is an appealing option because of its adaptability, flexibility, low cost, and high-yield production [84,85]. In a study, CdS NPs were produced by the wet chemical method by stirring 1 mM of CdCl_2_ with 5 mM sodium citrate, and then 1 mM of Na_2_S was added. The precipitate was twice rinsed with double-dH_2_O before being dried at 60 °C in the air [38].

Similarly, Harish et al. produced the CdS NPs by using the wet chemical technique. CdCl_2_ and Na_2_S were taken in a beaker at equimolar concentrations (0.1 M), and they were rigorously stirred for an hour. The production of CdS was indicated by the solution turning yellow [25].

#### 2.2.3. Solvothermal Synthesis

The solvothermal approach is one of the most effective processes for creating CdS NPs. In this procedure, precursors react in the presence of a solvent in a closed system at a temperature more significant than the boiling point of the solvent. The processing parameters of the reaction system, namely the types of solvents and reactants, temperature, and time, can be adjusted to regulate the size and shape of the product [86].

In a study, the solvothermal approach was used to create the CdS NPs. Concisely, a mixture of dH_2_O and 100% ethyl alcohol (1:3) was used to dissolve a certain amount of sodium hydroxide. Then, oleic acid was poured dropwise, and after stirring the mixture, CdCl_2_.5H_2_O (0.1 M) and Na_2_S·9H_2_O (0.1 M) aqueous solutions were added. After 10 min, it was placed in an autoclave to react at 180 °C for 12 h. After being cooled to RT, the yellow precipitate was extracted by centrifuging, and then it was carefully cleaned three times with 100% ethyl alcohol. Finally, to create CdS ink, the wet product was immediately distributed in 20 mL of toluene [51].

#### 2.2.4. Chemical Reduction Method

Chemical processes are the most practical and repeatable ways to create nanocrystals with precise control over size and structure. For this aim, chemical reduction or solution synthesis methods are frequently used [87].

A study used in situ chemical reduction to create nanocomposite films based on the polyvinyl alcohol (PVA)/CdS polymer. PVA has received much interest because of its exceptional mechanical and optical characteristics over a wide temperature range. PVA contains methane carbons with hydroxyl groups attached, which can be a source of hydrogen bonding and facilitate the production of inorganic NPs inside the PVA substance. Cd (NO_3_)_2_ and sodium sulfide nonahydrate (Na_2_S.9H_2_O) were prepared separately in 5 mL of dH_2_O at various molar concentrations (0.01, 0.02, 0.03, and 0.04 M) as sources of cations (Cd^2+^) and anions (S^−2^), respectively. The PVA solution serves as a stabilizer and a capping agent to prevent aggregation of the nano-CdS nuclei formed by the reaction of the dissolved Cd^2+^ ions with the released S^−2^. By adding a few drops of nitrate solution, the colorless PVA solution rapidly turned to yellow-orange, matching the color of the CdS [52].

#### 2.2.5. Thermal Decomposition Technique

Compared to using several reactants, using molecular precursors is better because it keeps the stoichiometry of finial products constant. The intermediate compounds produced during the thermal degradation of molecular precursors serve as capping agents and assist in synthesizing NPs with distinctive morphologies [53].

For the first time, a study examined how thermal degradation of a single source precursor, bis(thiourea)cadmium chloride, in several solvents led to the formation of CdS NPs with various morphologies. Thermal degradation of bis(thiourea)cadmium chloride in various pure and mixed solvents at 200 °C was used to create CdS NPs. In a 50 mL round-bottom flask, 10 mL of each of the solvents and the cadmium complex powder (1 mM) were added. The mixture was refluxed for 60 min at 200 °C, and the resulting slurry was precipitated with methanol [54].

In a study, the four distinct cadmium thiourea complexes were thermally decomposed at 200 °C in diphenyl ether to create CdS NPs. A certain amount of diphenyl ether (DPE) was mixed with 1 mM of each powdered cadmium thiourea complex. For 60 min, the mixture was refluxed in the air at 200 °C. Bis(thiourea)cadmium acetate, bis(thiourea)chloride, and bis(thiourea)nitrate, which correspond to brown-, bright-orange-, and yellow-colored CdS nanopowders, respectively, were produced [53].

#### 2.2.6. Sol–Gel Method

The sol–gel method offers several advantages, including simplicity, ease of handling, and low processing costs [57]. Arya et al. used the sol–gel method to synthesize CdS NPs in an aqueous medium. Maltose was employed as a capping agent along with cadmium nitrate (Cd(NO_3_)_2_), Na_2_S, and cadmium nitrate as a source of cadmium ions (Cd^2+^) and sulfide ions (S^2-^). The 0.5 M cadmium nitrate solution was heated to 50 °C. Then, Na_2_S solution was added dropwise. The mixture progressively turned from clear to pale yellow as soon as the reaction began, and when it was finished, it turned from pale yellow to dark yellow. To the solution mentioned above, maltose was added as a capping agent [57].

Another study created pure and Ni-doped CdS NPs by using the sol–gel method. The stoichiometric amount of Cd(NO_3_)_2_.4H_2_O, nickel nitrate (Ni(NO_3_)_2_.6H_2_O), and Na_2_S was dissolved in dH_2_O. Ammonia solution was added to the solution to change the pH. The precursor’s aqueous solution was thoroughly mixed for 4 h at 80 °C. As Ni-doped cadmium sulfide NPs developed, the translucent liquid progressively changed to a yellowish-orange color [58].

#### 2.2.7. Sonochemical Method

Recently, the sonochemical process has emerged as a valuable technique for creating new materials. The chemical impacts of ultrasound cause acoustic cavitation, which involves the production, development, and implosive collapse of bubbles in the liquid and produce transitory temperatures up to 5000 K, pressures of 1800 Mpa, and cooling rates exceeding 1010 K/s. Due to several benefits, including quick response times, manageable reaction conditions, and the capacity to create exceptionally pure NPs, this approach is the focus of many studies that are expanding quickly. As a result, this technique has been widely employed to create a variety of nanostructures [60].

In a study, the precursors CdCl_2_ and Na_2_S_2_O_3_ were dissolved in ethylene glycol (EG), along with the surfactant cetyltrimethylammonium bromide (CTAB), in the proper quantities to create nanoscale CdS. The starting solutions had cadmium and sulfur in molar ratios of 1:2, 1:4, 1:6, and 1:8. The initial solutions were subjected to high-intensity ultrasound irradiation under ambient air for 15 min, with the power set at 80 W. Yellow precipitates were produced after the completion of each reaction [88].

Similarly, the synthesis of CdS NPs by the sonochemical method, using a sonochemical bath at RT, was reported in another study. For this purpose, 0.1 M Na_2_S and 0.1 M cadmium acetate were mixed, with tryptophan serving as the chelating agent. The concentration of the chelating agent is crucial in regulating the size of NPs. The conical flask was placed in a sonochemical bath at RT after the three components had been well mixed. Tryptophan was employed in three different concentrations: (I) 0.1 M, (II) 0.2 M, and (III) 0.3 M. Then, for 60 min, ultrasonography irradiated the various solutions. Following irradiation, the yellow-colored suspension was centrifuged, and after 6 h of drying at 80 °C in the oven, the product was achieved [62].

#### 2.2.8. Combustion

The method of combustion provides a lot of benefits, including a reduction in processing time, energy savings, great purity, and the ability to produce small particles. However, the combustion approach was rarely used to create CdS–graphene oxide (CdS-GO) or -graphene composites in earlier investigations [89]. Using Cd(NO_3_)_2_, thiourea, and graphite as starting ingredients, researchers developed CdS/reduced graphene oxide composites by using a straightforward one-pot combustion technique. In a study, GO was created using the modified Hummers’ method. In contrast, the CdS/reduced graphene oxide (CRG) composites were created using a combustion technique with thiourea as the fuel and Cd(NO_3_)_2_ as the oxidant, respectively. An aqueous mixture of Cd(NO_3_)_2_ and thiourea was treated with ultrasonic radiation for 10 min to create a transparent solution with a mole ratio of 3:5. After adding the proper amount of GO to the abovementioned solution, a 30-minute ultrasonic treatment was performed, and a jelly-like consistency was created. The CRG composites were produced due to the exothermic redox reaction between nitrates and thiourea [89].

Another work produced Mn^2+^-doped CdS (MnxCd1xS: x = 0.0, 0.3, and 0.5) nanocrystallites, using a quick and effective microwave-assisted combustion technique. Various molar ratios of Mn^2+^ (MnxCd1xS: x = 0.0, 0.3, and 0.5) were added to CdS to create the samples. Then dH_2_O was used to dissolve stoichiometric amounts of Cd(NO_3_)_2_, manganese nitrate, and thiourea before being put into a silica crucible and heated in a home microwave (2.45 GHz, 800 W). The solution initially boils, and then it dehydrates and decomposes with the development of gases. The solution rapidly vaporizes and turns into a solid once it reaches the level of spontaneous combustion. The production of CdS nanocrystallites from the reaction of Cd(NO_3_)_2_ salts and thiourea in the solution is generally thought to have one plausible pathway. In an 800 W microwave oven for 10 min, the entire microwave-assisted combustion technique yields CdS nanocrystallite powders [90].

#### 2.2.9. Micro-Emulsion Method

An efficient method for producing a variety of mono-dispersion NPs of various sizes and shapes is micro-emulsion (ME) [91]. MEs are essentially systems of two immiscible liquids that are isotropic, surfactant-stabilized, and thermodynamically stable. Since the surfactant-stabilized droplet phase can be thought of as a “nanoreactor” for synthesis, MEs are perfect for the creation of NPs. Due to surface tension, all nanoreactors are spherical and, after some equilibration time, have the same size [92].

In a study, CdS NPs were produced using the microemulsion method in AOT (sodium bis (2-ethyl hexyl) sulfosuccinate), water, and n-heptane. Using the ternary phase diagram between AOT, water, and n-heptane, the transparent phase was discovered. Because the microemulsion solution is stable, this clear phase area must be prepared. Firstly, they put Sample A (AOT, water, and n-heptane) in the ternary phase diagram. Using dissolved water and salt Cd(NO_3_)_2_.4H_2_O for Sample B and Na_2_S.8H_2_O for Sample 3, the same procedure was repeated. Then, a clear plot was plotted in the ternary phase diagram during the apparent phase. A microemulsion solution was created by diluting Samples B and C with a similar concentration in a glass vial. N-Heptane was employed to dilute the NP from the ME solution [63].

In an ME under ultrasound, Entezari et al. created semiconductor CdS NPs (with a diameter of about 2 nm) with a hexagonal phase, at a comparatively low temperature (60 °C) and in a short time. Initially, a certain amount of sulfur was dissolved in heated p-xylene. Then, a quaternary oil-in-water (O/W) ME was created using the correct ratios of CTAB, 1-butanol, p-xylene, and water as the reaction medium for the synthesis of CdS NPs. Two independent portions of the ME A = 80% and B = 20% *w*/*w* were created. The B component of the ME comprised ethylenediamine (0.41 M) and CdCl_2_ (0.025 M) in the aqueous phase, while the A portion of the ME contained sulfur (37.8 mg) in the oil phase. A transparent ME of B was combined gradually with a clear ME of A. The combined ME was stirred individually and heated for 30 min at 60 °C. In a further experiment, the ME combination previously indicated was exposed to ultrasound for 30 min. By turning off the circulating bath, the temperature was raised from 30 to 60 °C during sonication. Immediately after the start of the nucleation processes, a high number of nuclei were created after 5 min [93].

### 2.3. Physical Methods

#### 2.3.1. Pulsed-Laser Ablation

A method to create colloidal semiconductor NPs is pulsed-laser ablation in liquid (PLAL). Numerous NPs, including noble metals, alloys, oxides, and semiconductors, are regularly made using PLAL [64]. As this technique is straightforward, does not require a surfactant, and allows for precise control over the size and form of the produced NPs, PLAL has gained much interest. The features of synthesized NPs can be affected by several factors in this method, including laser fluence, laser wavelength, pulse duration, and colloid solution type [65].

In a study, the synthesis of CdS NPs by Nd:YAG laser ablation of CdS target in methanol was reported without employing any surfactant. For this purpose, the pellet was first put at the bottom of a glass vessel that contained 5 mL of pure methanol, without any surface-active agents. The laser energy used for the ablation procedure was between 100 and 500 mJ per pulse, with a 15-minute ablation time, under normal pressure, in the open air. Using an optical microscope, the laser beam diameter on the surface of a CdS pellet was determined to be 2.37 mm. The XRD and SEM analysis showed that the NPs possess a single-phase hexagonal structure of CdS, with laser fluence controlling their morphology and size [65].

In other research conducted by Goncharova et al., dH_2_O, ethyl alcohol, ethyl acetate (ETAC), and methyl methacrylate (MMA) were used for laser ablation. The purity of the metal zinc target, which had dimensions of 40×20×5, was 99.5%. The sulfide precursors included hydrogen sulfide (H_2_S) and thioacetamide (TAA) CH_3_CSNH_2_. The precursors were initially introduced into initial solvents before laser ablation. The Cd content in the solution following ablation was 10–30 times higher than the precursor concentration when converted to sulfur atoms. Through the lateral surface of the cylindrical glass reactor, a quartz lens (F = 30 mm) focused laser radiation onto the surface of the target. Such a radiation input plan allowed for steady-state focusing and prevented solution splashing. On the sample surface, the radiation pulse power density ranged from 0.5 to 1 GW/cm^2^. The target was shifted automatically in the XY plane to ensure uniform irradiation. Based on the reduction in the target mass, the concentration of NPs in the solution was calculated and was reduced to Cd in all measurements at 0.15–0.20 g/L [66].

#### 2.3.2. Hydrothermal Method

One of the most popular techniques for creating nanomaterials is hydrothermal synthesis. The hydrothermal approach involves using an aqueous solution (water) as a reaction system in a particular closed reaction vessel, where the temperature of solvents can be raised via heating in conjunction with autogenous pressures to approximately their critical points. A substance that is weakly soluble or insoluble under normal circumstances is dissolved and recrystallized by this process [94,95]. The following three components make up the process: precursor that comprises reactants in the form of solutions, gels, or suspensions; mineralizers, which are organic or inorganic additives used at high concentrations to regulate the pH of solutions. Additives were organic or inorganic and applied at fairly low concentrations to enhance particle dispersion or control crystal shape [95]. As mentioned above, it is a solution-reaction-based method, and nanomaterial synthesis can take place over a wide temperature range, from RT to extremely high temperatures. According to the vapor pressure of the primary components in the reaction, either low-pressure or high-pressure conditions can be utilized to control the morphology of the materials. There are new synthesis techniques, including template-free, self-assembling, catalytic synthesis, and microwave-assisted hydrothermal synthesis [96]. Hydrothermal synthesis presents some benefits: being very pure, with a regulated morphology; having a limited size distribution and comprising single crystals; having a high powder reaction rate; having good dispersion in liquid; and being almost pollution-free. What is more, the prepared powder is not conglomerated but is fine-grained, and it does not need high-tech equipment [67].

Crystallization directly from the solutions is a process used in the hydrothermal method. It typically involves two steps: crystal nucleation and growth. When the solubility of a solute in a solution goes above what is considered to be the maximum, nucleation results. The process is irreversible, and the solute precipitates into crystal clusters that have the potential to reach macroscopic size. After nucleation, the crystals grow sequentially or simultaneously through a series of procedures, including the addition of growth components from the bulk solution into the already-existing crystal entities and leading to greater sizes. Growth units might have the same or distinct structures as crystal entities, while sharing the same composition [95].

In a study, the hydrothermal technique was used to create CdS NPs with a diameter of 50.8 nm. Three steps were performed in the hydrothermal procedure. The first step involved adding a certain amount of cadmium acetate dehydrate Cd(CH_3_COO)_2_.2H_2_O as a source of Cd to dH_2_O and stirring for 1.5 h at 60 °C. The second stage included 1.5 h of stirring at 60 °C while an equal amount of sodium sulfide is dissolved in dH_2_O. Moreover, in the final stage, the mixture from stage two is gradually added to the mixture from stage one. The solution temperature was kept at 60 °C and agitated for 3 s. The prepared combination was then placed in a Teflon-lined sealed stainless-steel autoclave and kept at 170 °C in the oven for 24 h. After slowly cooling down to RT, the final product was rinsed with dH_2_O multiple times, using an electrosonic machine; it was then lifted to dry at 50 to 60 °C overnight. Finally, it is possible to collect the yellow powder [67].

Zhong et al. prepared CdS NPs by a hydrothermal synthesis method. First, solutions of sodium sulfate and Cd(OAc)_2_ were separately prepared in dH_2_O. The two solutions were then mixed, magnetically stirred for 24 h, and stored for 24 h. Suction filters were applied to the solution, and the wet powders were then added to dH_2_O and moved to a hydrothermal reactor, where they were heated for 72 h at 473 K. The yellow powders were filtered and then rinsed with dH_2_O and ethanol, filtered, and kept in an oven at 363 K for 24 h [69].

#### 2.3.3. Microwave-Assisted Method

A recent and quickly evolving technique for synthesizing NPs is a microwave-assisted method [72]. Since these types of methods utilize fewer solvents, are quick and highly efficient, and can produce NPs with controllable morphologies, they have drawn much attention [73]. Furthermore, microwave synthesis can create tiny particles with a narrow particle size distribution and good purity while requiring a significantly shorter reaction time than conventional techniques. Solvents can considerably affect the size and morphology of the resulting products while they are being formed into NPs under microwave irradiation. The pace of heating, the temperature of the reaction, and the collision between reactant molecules vary in different solvents [74].

In a study, a precursor solution, including cadmium–acetate and thioacetamide, was used to produce CdS NPs, using a microwave-assisted technique. A newly made aqueous solution of cadmium acetate (1 M) and thioacetamide was used in this experiment. Thioacetamide (CH_3_CSNH_2_) was stoichiometrically combined with a solution of cadmium acetate (Cd(CH_3_COOH)_2_.2H_2_O). To obtain the precipitated bright-yellow-colored CdS NPs, the solution was further microwaved with a power of 900 W after adding a few drops of NaOH. For the following 6 h, the CdS NPs were dried at 1000 °C in an oven. The resulting bright yellow CdS NPs were examined for their structural, optical, and morphological characteristics [70].

Another study created CdS NPs by using a microwave-assisted hydrothermal process. To achieve this, (NH_2_)_2_CS, C_4_H_9_OH, C_6_H_12_, and cetyltrimethyl ammonium bromide (CTAB) were uniformly combined to make a suspension. CdSO_4_.8H_2_O solution was then added dropwise, and the resulting solution was continuously stirred for 30 min till the suspension was developed. The suspension was then placed in a microwave reactor and heated to 150 °C for 10 min. Finally, it was allowed to dry naturally [73].

The common polyol procedure is dissolving a metal precursor (such as silver, cadmium, etc.) together with a stabilizer or protective agent in a polyol medium [71,74,97]. EG is the simplest member of the polyol family, chemically speaking. The polyols consist of two major families of compounds based on EG. Diethylene glycol (DEG), triethylene glycol (TrEG), tetraethylene glycol (TEG), and so on, up to polyethylene glycol, are all included in the first group (PEG). In contrast, pentanediol (PD), propanediol (PDO), butanediol (BD), and other diols are included in the second group [98].

A study used cadmium chloride and thioacetamide as the cadmium and sulfur sources, respectively, to create CdS NPs employing a quick and easy microwave-assisted polyol approach. In a typical synthesis, 100 mL glass beakers containing 20 mL of EG received 5 mM of cadmium chloride and 5 mM of thioacetamide, and they were stirred for 15 min. The precipitates were centrifuged before being thoroughly cleaned and rinsed with dH_2_O and ethanol [74].

**Figure 2:**
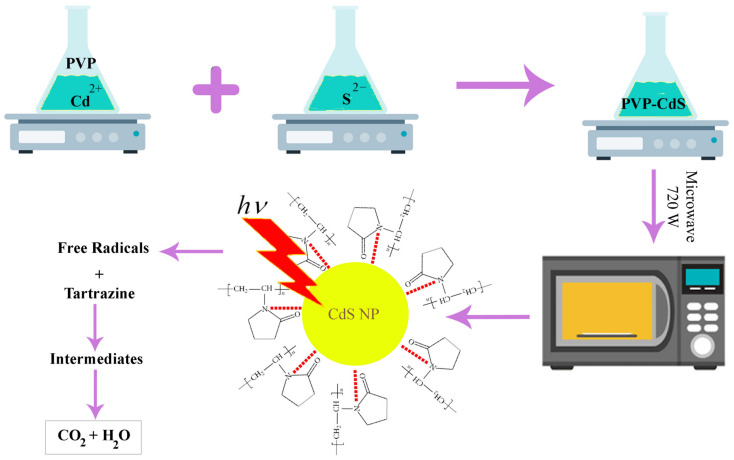
Microwave-assisted polyol synthesis of PVP-capped CdS nanoparticles for the photocatalytic degradation of tartrazine.

According to a usual protocol, specific amounts of cadmium chloride 2.5-hydrate and polyvinylpyrrolidone (PVP) were individually mixed in 30 mL and 20 mL of EG, respectively, and then heated for 60 min, at 75 °C, with stirring. The solutions were gradually combined and heated at 100 °C for 40 min, with stirring. In parallel, 20 mL of EG was used to dissolve thiourea, which was then slowly added to the hot (PVP-Cd^2+^) solution after being heated for 100 min, at 75 °C, with stirring. Within 45 min, the temperature of the new combination was raised progressively from 16 to 170–180 °C, and it was maintained at this temperature until the majority of the solvent had evaporated. As CdS began to develop at this point, the solution’s color changed from bright yellow to a dark orange suspension. The suspension was then microwaved for 5 min (720 W, 50% duty cycle). Finally, black crystallites of PVP-capped CdS NPs were produced after the resulting powder was calcined in a furnace for 120 min, at 450 °C (Figure 2) [71].

#### 2.3.4. Reflux Method

The aqueous-based reflux method is a straightforward, inexpensive procedure that yields the desired product with excellent control of reaction variables. This technique involves continuously heating the reaction solution to provide the energy required for the reaction. By changing variables such as the reaction duration, precursor concentration, and solvent types, it is possible to adjust the size, morphology, and crystallinity of the materials. To achieve the required phase and morphology of the nanostructures, the variables, including the sequence of precursor addition, the period of the reflux, and the cooling rate, should be tuned [99].

Poornaprakash et al. used a simple reflux method to create CdS, CdS:Er (2 at%), and CdS: Er (4 at%) NPs. Aqueous cadmium acetate solution was dissolved in dH_2_O, while stirred, to create pure CdS NPs. Then, dropwise additions of Na_2_S aqueous solution were made to the abovementioned cadmium solution. Polyethylene glycol was then added to serve as a capping agent. At 30 °C, the resulting yellow suspension underwent continuous stirring for 15 h [75].

### 2.4. Physicochemical Method

#### Mechanochemical Processes

Mechanochemical processing has become a viable technique in recent years for the synthesis of many nanomaterials (such as three-dimensional (3D) metal–organic frameworks). In mechanochemical synthesis, mechanical force is used to carry out chemical reactions. Mechanochemistry works by milling or grinding solid reactants together, using several effective techniques, such as liquid-assisted grinding (LAG), ion- and liquid-assisted grinding (ILAG), film grinding, or grinding–annealing [100].

**Figure 3:**
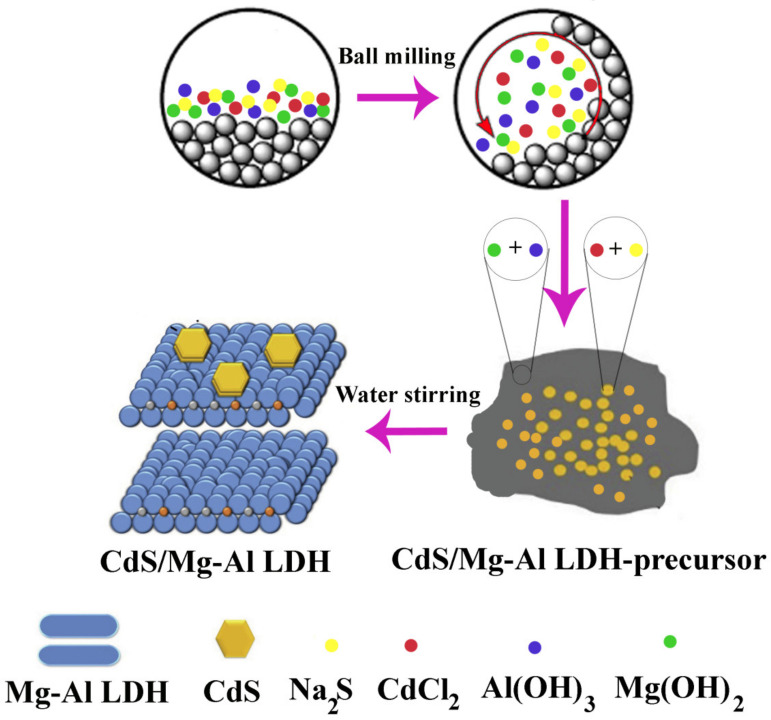
Schematic illustration of ball-milling approach for the synthesis of CdS/Mg-Al LDH as photocatalysts.

In a study, CdS/Mg-Al LDH-precursor composites were created using a mechanochemical technique. To do this, CdCl_2_ and Na_2_S were combined and processed in a planetary ball mill. After grinding, pure CdS was added to dH_2_O, stirred for 2 h, and then filtered. Following the complete removal of the sodium impurities from the product, the solid residue was rinsed and dried in a vacuum dryer at RT (Figure 3) [76]. Furthermore, through a two-step solid-state mechanochemical synthesis, Baláž et al. created CdS/ZnS nanocomposites. The milling procedure was carried out in two steps to create CdS/ZnS nanocomposites. In the first step, CdS was produced by 15 min of milling a stoichiometric combination of (CH_3_COO)_2_Cd.2H_2_O and Na_2_S.9H_2_O. In the second stage, the milling pot was filled with a stoichiometric combination of (CH_3_COO)_2_ Zn.2H_2_O and Na_2_S.9H_2_O, and the already created CdS was milled for an additional 15 min. This process formed CdS/ZnS nanocomposites [77].

## 3. Characterization Methods of CdS NPs

**Figure 4:**
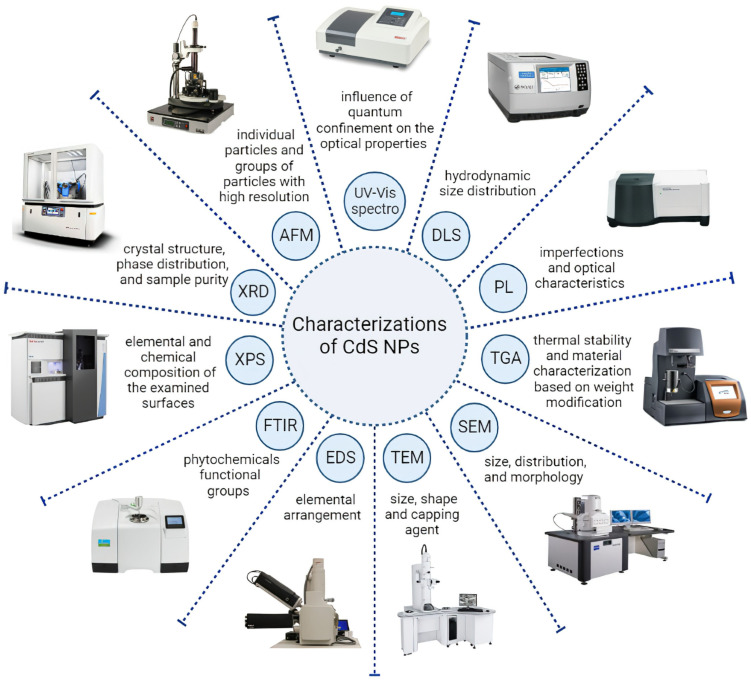
The characterizations methods of CdS NPs and their main applications.

The development of nanotechnology through various approaches in diverse research areas has led to the requirement to use analytical techniques for the physicochemical analysis and characterization of NPs [101]. The basic properties that depend on the characterization of CdS NPs are generally size, shape, and surface charge, which depend upon the method employed for the synthesis [15]. The CdS NPs synthesized by diverse methods have various sizes, shapes, and physicochemical properties and are characterized with the help of techniques such as UV–visible spectroscopy (UV–Vis spectra), Fourier-transform infrared spectroscopy (FTIR), photoluminescence (PL), dynamic light scattering (DLS), energy-dispersive spectroscopy (EDS/EDAX/EDX), powder X-ray diffraction spectroscopy (XRD), scanning electron microscopy (SEM), transmission electron microscopy (TEM), atomic force microscopy (AMF), X-ray photoelectron spectroscopy (XPS), and thermal gravimetric analysis (TGA) summarized in Figure 4. Moreover, the recent study of CdS NPs and the characterization methods are shown in Table 1.

UV-Vis spectroscopy is an efficient technique to conceive the influence of quantum confinement on the optical properties of NPs [28]. Moreover, UV–Vis spectra were highly used in the optimization of the concentration of the chemical agents, time, and temperature of CdS NPs’ synthesis reaction [28,32,33,34,41,42,56,102]. Sankhla and colleagues conducted a study in which they synthesized CdS NPs by incubating the precursor salts with *E. coli*. They captured the UV-Vis spectrum at various time intervals, including 2, 4, 6, 8, 12, and 24 h. The highest absorption was obtained in the 24-hour sample. In the region below 450 nm in the spectrum, they observed a distinct absorption peak at 400 nm. This noticeable blue shift in comparison to bulk CdS at 515 nm is the feature of the quantum size domain for these NPs. The prominent peak at 400 nm is attributed to the optical transition of the first excitonic state. Because the size of the NPs gets smaller, the wavelength of maximum exciton absorption typically gets smaller as a result of the photogenerated electron–hole pairs’ quantum confinement [28]. Haq Bhat et al. acquired UV-Vis spectra from a reaction mixture containing CdS NPs that were synthesized using *Panicum sarmentosum*, both before and after a 12-hour incubation period. The CdS NPs had absorption maxima between 300 and 400 nm wavelength ranges, which were connected to the surface plasmon resonance band, according to the UV-Vis spectra obtained after 12 h. This finding indicates the existence of a blue shift from the normal absorption maximum of bulk CdS, which occurs at approximately 347 nm [42].

FTIR is a type of vibratory spectroscopy that is useful for investigating the structural properties of NPs [101]. The FTIR investigation was utilized to understand the nature of diverse phytochemical functional groups responsible for NPs formation [41]. Shivashankarappa and colleagues synthesized CdS NPs by using *Bacillus licheniformis* and a reaction between cadmium chloride and sodium sulfide. Four different ratios of the compounds (1:1, 2:1, 3:1, and 4:1) were used to investigate the impact of cadmium chloride on NPs’ synthesis. The FTIR analysis of the synthesized NPs showed various absorption bands in the range of 400–4000 cm^−1^. The absorption peaks of the different ratios were observed at different wavenumbers, indicating the presence of distinct functional groups or chemical bonds in the synthesized NPs. The absorption peaks at different ratios of cadmium chloride and sodium sulfide were observed at distinct wavenumbers: the 1:1 ratio had absorption peaks at 3335.28, 2928.38, 2861.84, 1645.95, and 1405.85 cm^−1^; the 2:1 ratio had peaks at 3322.75, 2928.38, 2860.88, 1645.95, and 1406.82 cm^−1^; the 3:1 ratio had peaks at 3324.68, 2928.38, 2856.06, 1647.88, and 1406.82 cm^−1^; and the 4:1 ratio had peaks at 3307.32, 2923.56, 2855.1, 1655.59, 1536.02, 1406.82, and 1006.66 cm^−1^. in all samples, the peaks in the range of 3300–3335 cm^—1^ were related to O-H and N-H stretching vibrations due to the presence of protein on the CdS surface. The presence of proteins was demonstrated by the prominent peaks at 1645–1655 cm^−1^, which were attributed to amide I and II. Peaks in the region of 2855 to 2928 cm^−1^ are caused by the alkanes’ C-H stretching vibrations. The findings demonstrated that, in comparison to all of the ratios, NPs synthesized with a 4:1 ratio had the highest number of functional groups [34].

X-ray diffraction analysis, as an adjustable, simple, and non-destructive method, was performed on the prepared CdS NPs to study the crystal structure and phase distribution, measurement of crystalline percentage, and analysis of sample purity [56,77]. As an example, the structure of the green synthesized CdS NPs using *Panicumsarmentosum* extract was confirmed by the characteristic peaks obtained in the XRD image. The XRD pattern of these NPs demonstrated four prominent peaks in the spectrum of 2θ values ranging from 0 to 100 [42]. Moreover, Sankar et al. demonstrated that the XRD pattern of sol–gel-prepared Ni-doped CdS NPs had peaks corresponding to Miller indices (100), (002), (101), (102), (110), (103), (200), (112), (201), (202), (203), (211), and (105) confirmed the hexagonal structure of CdS NPs. Furthermore, the XRD analysis of Ni-doped CdS NPs showed no impurity peak, except for Ni particles, which confirmed the high purity of the prepared NPs [56].

The zeta potential is a significant parameter comprehended to influence the long-term stability of colloidal dispersions. It shows the magnitude and nature of stability referring to surface charge, which is essential in determining the interactions of cell membranes. The zeta potential of NPs is employed to predict the increase in susceptibility of aggregate formation when NPs interact with biomolecules [28,101]. Harishet al. synthesized uncoated CdS NPs and chitosan-coated CdS NPs by wet chemical synthesis. The zeta potentials of uncoated CdS NPs and chitosan-coated CdS NPs were +5.3 mV and +20.2 mV, respectively. In this study, the increase in positive surface charge was attributed to the chitosan adherence to the surface of CdS NPs [25].

The size, size distribution, and morphology of the NPs can be analyzed directly by SEM. Moreover, the purity of the NPs and their degree of aggregation can be obtained by this technique. However, there is a possibility of sample destruction during the preparation process in this technique, which may lead to false results [101]. The various morphologies of CdS NPs, including spherical [43], rod shape [65], pyramid-like [54], and even irregular particle shape [51], were obtained from the SEM technique.

High-resolution TEM (HR-TEM) is an imaging strategy that allows imaging of the crystallographic form of NPs at the atomic scale [33,43,56]. For example, Sandoval-Cárdenas et al. demonstrated the dispersed individual spheroid CdS NPs with diameters between 7 and 15 nm. Moreover, the HR-TEM image of this study revealed the presence of planes, manifesting the presence of a crystalline structure [32]. Furthermore, the HR-TEM image of the Sankar et al. study confirmed the porous spherical clustered nano-assemblies during sol–gel synthesis of Ni-doped CdS NPs [56].

AFM is employed to visualize individual particles and groups of particles with high resolution upon sample scanning at the submicron level by the atomic-scale probe tip. The final result of this technique is a topographical map based on the forces of the model surface [39,101]. Abd et al. synthesized the CdS NPs via the pulsed laser ablation method and analyzed the surface morphology of CdS NPs by AFM. The outcomes revealed that the NPs covered the surface of the substrate nicely and with a uniform distribution. Moreover, the image obtained by AFM analysis demonstrated that synthesized NPs include small particles arranged with hemispherical forms with monopod rods [65].

DLS is the most common technique to study hydrodynamic particle size and distribution of the particles over a spectrum of sizes. The hydrodynamic diameter of the particles is influenced by the materials employed in synthesizing the particles and is usually more prominent than the FESEM and TEM analyses [36,103,104].

PL is a sophisticated method for assessing the properties of imperfections and optical characteristics of CdS NPs [56,67]. Sankar et al. performed the PL technique for sol–gel-prepared Ni-doped CdS NPs with an excitation wavelength of 325 nm at RT. Their results showed two emission peaks: the peak at 330–342 nm was due to exciton recombination via an exciton-exciton collision procedure, and the weak peak at 450–459 nm is usually attributed to close bar border emission due to free exciton recombination. In addition, it was found that increasing the Ni resulted in a decreased intensity of PL [56].

XPS was utilized to quantify the elemental and chemical composition of the examined surfaces by using the excitation of X-rays [30,101]. For instance, The Cd, S, O, C, and P elements are observed on the surface of green CdS NPs by XPS analysis. Moreover, the surface of the fabricated CdS NPs was highly oxidized, as demonstrated by the relatively high-density O-associated XPS (O1s) [30].

EDS is an X-ray technique used to detect the elemental arrangement of NPs [41,57]. The EDX pattern of plant-mediated synthesized CdS NPs in the study by Ullah et al. revealed intense peaks of Cd and S that are their elemental signals from the emission of energies, respectively [41]. Moreover, Arya et al. accomplished EDS for prepared CdS NPs. The EDS spectrum revealed the peaks that belong to Cd and S, which indicate the purity of the synthesized CdS NPs [57].

TGA is a method to assess thermal stability and material characterization based on weight modification. This technique can determine the amount of weight modification of a material, either as a function of rising temperature over time or isothermally as a function of time, in an atmosphere of some gases, such as nitrogen [42]. The CdS NPs obtained by chemical precipitation reactions demonstrated high thermal stability, with a high melting point and the absence of any contaminant. The eventual results of TGA revealed suitable thermal stability up to 700 °C. Based on this result, Qutub et al. reported that the synthesized CdS NPs can be employed as a pigment in paints and engineered plastic [44].

## 4. Biomedical Applications

**Figure 5:**
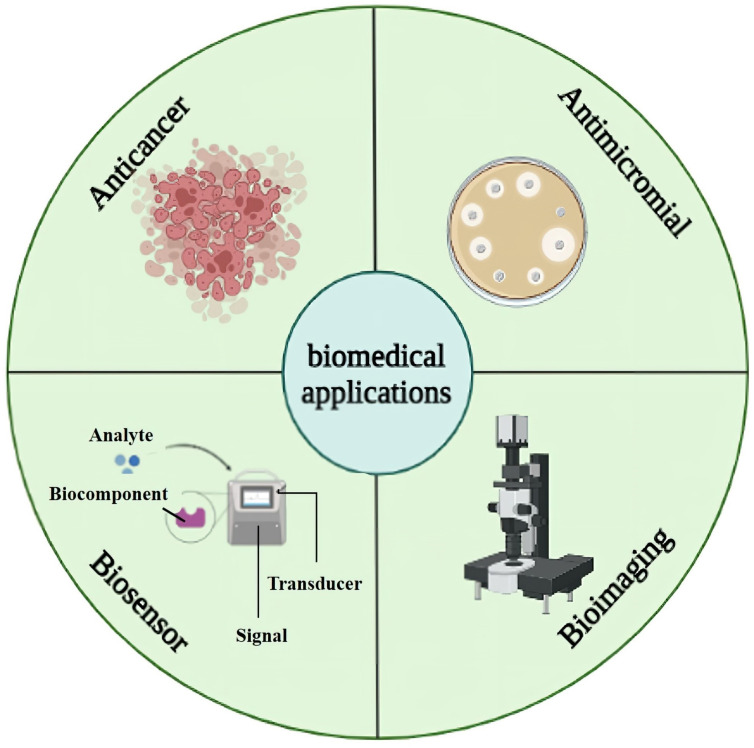
Summary of biomedical applications of CdS NPs.

NPs have the potential to be used in biomedicine as a tool for imaging, drug delivery, diagnostics, and therapy. The exceptionally controlled luminescence, continuous excitation spectrum, narrow emission bands, and ease of functionalization for targeting by CdS NPs have led to a wide range of biomedical applications of CdS NPs, which are frequently employed in drug delivery, molecular pathology, bioimaging, and biosensor applications [42,105,106]. Moreover, bioconjugates, including DNA, proteins, and monoclonal antibodies, can be purposefully attached to CdS NPs for employment as a bioimaging agent and drug delivery [25]. In addition, the non-toxicity of CdS NPs has led to their use as drugs and diagnostic tools in vivo and in vitro models [107]. Here, we assessed the most effective uses of CdS NPs in medicine, including bioimaging, biosensors, anticancer, and antimicrobial effects, which are shown in Figure 5.

### 4.1. Anticancer Activity

CdS NPs vary in toxicity depending on their size, chemical constituents, and coating. NPs are thought to have high cytotoxic potential due to their unique characteristics, such as their small size, high surface-to-volume ratio, and gradual release capabilities. Cell death could have happened due to reactive oxygen species (ROS) generation or the release of the internal cadmium ion (Cd^+2^) from CdS NPs into the cell medium. The surface oxidation of the CdS NPs causes the release of cadmium ions from the CdS NPs into the cell medium [36]. To affect cancer cells and induce cell toxicity, CdS NPs cause oxidative stress, inhibit antioxidant function, exert genotoxic effects, affect calcium homeostasis in cells, and induce apoptosis [108]. In addition, CdS NPs generate ROS by electron–hole pairs to transfer electrons to oxygen, directly interact with the intracellular antioxidant system, or elevate the ROS molecules by releasing Cd^2+^ ions [23].

The study by Apykhtina et al. showed an apparent dose-dependent cytotoxic effect of CdS NPs on the MEC, EK-293, and IMR-32 cultured cell lines, resulting in decreased membrane permeability, lysosome activity, and mitochondrial function, as well as reduced protein synthesis. The “DNA comet” experiment shows that, in comparison to 9–11 nm NPs, CdS NPs in the size range of 4–6 nm showed a more severe genotoxic effect [108].

In another study, Shivashankarappa et al. used *Escherichia coli* to produce CdS NPs biologically. The cytotoxic effect of synthesized CdS NPs was investigated on Mus musculus skin melanoma (B16F10) and human epidermoid carcinoma (A431) cell lines. The results demonstrated significant inhibition of 75.71% at a concentration of 0.2 mM against B16F10 and 81.53% at a concentration of 0.1 mM against A431 cell lines. The inhibition was compared to a common anticancer medication called 5-aminolevulinic acid (5-ALA), which inhibited B16F10 cells by 31.95% and A431 cells by 33.45% at a dose of 1 mM. The outcomes of this study demonstrated that CdS NPs had superior cytotoxic action against cells compared to traditional anticancer drugs [23].

**Figure 6:**
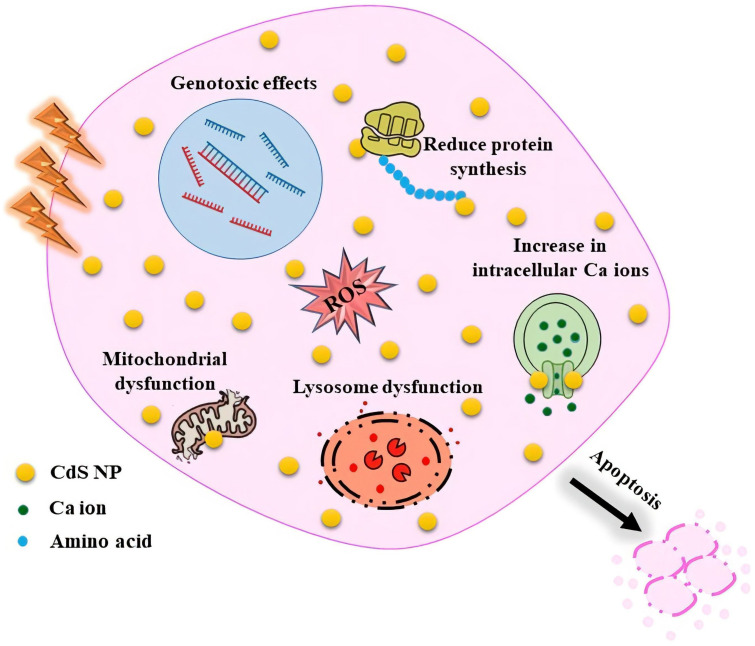
Summary of the effective mechanisms involved in the anticancer activities of CdS.

The recent anticancer experiments with CdS NPs are displayed in Table 2. These NPs generally exhibit effective anticancer activities, which appear to be connected to ROS generation and NPs’ cell penetration. A summary of the effective mechanisms involved in the anticancer activities of CdS NPs is shown in Figure 6.

### 4.2. Antimicrobial Activity

Today, much attention has been paid to the antimicrobial activities of nanoparticles, especially in the case of microorganism resistance [113]. Smaller NPs typically contain more surface atoms, giving them a larger surface area for contact with bacteria. As a result, smaller NPs are more active than larger NPs [23]. The type of cell wall structure, which governs microbe permeability, could cause variation in microorganism susceptibility. The outer membrane of Gram-negative bacteria contains lipopolysaccharides, which prevent the entry of macromolecules and other hydrophilic compounds. In contrast, Gram-positive bacteria either do not produce lipopolysaccharides or do so in minimal amounts. The cell walls of these bacteria also have numerous peptidoglycan layers and negatively charged glycerin chains (teichoic acid). Upon interacting with the negative charge of the cell wall, cadmium may rupture the cell wall. Moreover, the bacterial-growth-signaling system may be impacted by NPs, which would impair the viability of the cell [36,114]. CdS NPs have significant contact with the thiol groups found in important bacterial respiratory enzymes, which is why they have an inhibiting effect on bacterial cells. The positive charge of CdS NPs form electrostatic interactions with the negative-charge proteins on the surface of bacterial cells as they travel through the membrane, altering the internal structure of bacteria cells [42]. When the thiol groups in proteins interact with the released ions from the NPs, ROS are created, which disrupt the cell structure and, subsequently, cell function. Upon binding CdS NPs to the protein layer, inhibition of active transport, dehydrogenase, and enzymatic activity occurs in the periplasm zone, thus inhibiting the synthesis of DNA, RNA, and proteins, followed by cell lysis [23]. Shivashankarappa et al. fabricated CdS NPs by using *Escherichia coli*. The synthesized CdS NPs had greater antibacterial and antifungal action than conventional medications when tested against several foodborne pathogens, including *Pseudomonas aeruginosa*, *Bacillus licheniformis*, *Escherichia coli*, and *Aspergillus flavus* [23].

Haq et al. used the plant *Panicum sarmentosum* to create CdS NPs. These CdS NPs synthesized with *Panicum sarmentosum* were discovered to be toxic to *Escherichia coli* and *Staphylococcus aureus*. It was discovered that the antibacterial property and the diameter of the bacterial inhibition zone increased with the concentration of NPs. Additionally, *Escherichia coli* was discovered to be more resistant to CdS NPs than Gram-positive bacteria (*Staphylococcus aureus*) [42].

**Figure 7:**
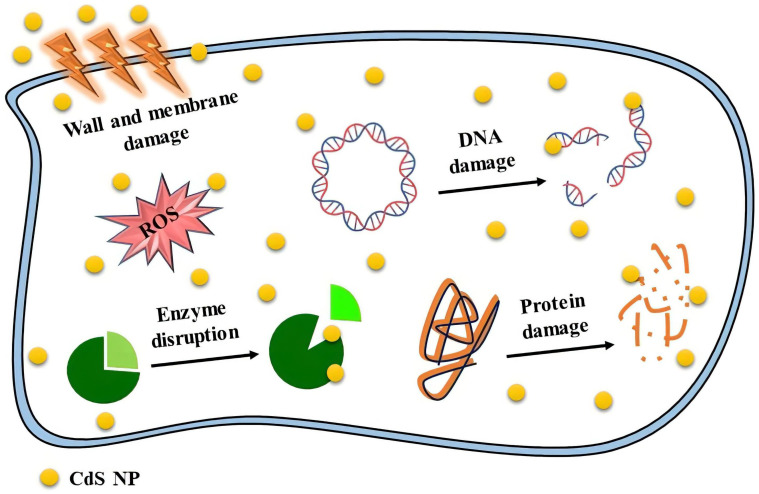
Summary of the mechanisms of action of the antimicrobial activities of CdS NPs.

Antimicrobial studies of CdS NPs conducted in recent years are shown in Table 3. These NPs show different antimicrobial properties, which depend on their characteristics, including their size and synthesis method. A summary of the effective mechanisms involved in the antimicrobial activities of CdS NPs is shown in Figure 7.

### 4.3. Bioimaging Application

Fluorescence imaging is an alternative modality that can provide real-time imaging with high contrast and resolution, but it has limitations in regard to tissue penetration. This imaging modality is beneficial for diagnosis in preclinical applications for tumor detection and brain injury. It has also been demonstrated that it is applicable in single-cell tracking for studying the immune system and circulating tumor cells, as well as fluorescence-guided surgery [118]. QDs are viewed as an excellent replacement for organic fluorophores due to their remarkable photostability, size-related absorption and diffusion, longer lifetime of the excited state, biocompatibility, broad absorption, and narrow emission bands [118,119]. Additionally, functionalized QDs are advantageous and may be altered with a variety of biomolecules and tiny biological polymers, which not only increase their bioactivity but also reduce their adverse effects. Due to these properties, QDs are highly effective at attaching to target cell membranes, making them excellent probes for cell imaging, diagnostics, and the delivery of therapeutic substances. To be employed as sensitive tools for biological targets, QDs may be covalently linked to several biomolecules in an aqueous solution. Accepted bioconjugation techniques could be used to achieve this, particularly for imaging and photodynamic therapy for cancer [105]. For in vitro cellular imaging, CdS is one of the most extensively studied fluorescent materials. However, the high level of toxicity restricts the use of CdS NPs in vivo [119,120]. The cell membrane does not allow all substances to enter the cell quickly due to the selective permeability of the cell membrane, but the NPs can easily enter the cell through pinocytosis and endocytosis [119].

Anna et al. demonstrated the potential of cadmium–zinc-sulfide QD azine-grafted onto halloysite clay nanotubes as bioimaging agents. Due to their excellent light-scattering qualities, halloysite nanotubes appear as bright spots when examined with enhanced dark-field microscopy. The cytoplasm and cell membrane are also visible; however, the halloysite-deficient areas of the cell seemed faint [120].

Gonzalez et al. used CdS QDs to target and image *HeLa* cells, which were capped with dextrin and bioconjugated with doxorubicin (DOX). These synthesized CdS NPs have a strong fluorescence emission in the green and red spectrum, which was helpful for fluorescence imaging on *HeLa* cells. According to the results, these synthesized CdS QDs accumulate slowly in the cytoplasm and nucleus of cells [105].

Some examples are listed in Table 4 for applications of CdS NPs in cell imaging. Due to the stability of the fluorescence and excellent penetration into the cell without changing the cell morphology, the images obtained by these NPs have high quality and resolution.

### 4.4. Biosensor Application

The sulfur-containing nanomaterials have excellent properties, such as nanometric scale, water-dispersibility, excellent catalytic activity, conductivity, biosafety, photo-activity, and fascinating optical properties, which make them useful in various biosensing applications. Metallic sulfide nanomaterials have been used as photoactive materials in biosensing systems to generate photocurrent stimulated by light [122]. CdS QDs are recognized as active fluorescent probes and photoelectrochemical materials for the detection of biological analytes. Moreover, CdS QD outperforms conventional fluorescent materials due to its photostability and broad range of excitation wavelengths [123,124]. CdS NPs are the most promising nanomaterials for coating electrodes because they have outstanding photocatalytic activity, significant energy gaps, and high electric conductivities [125]. Due to their functional groups (e.g., amino, carboxyl, and sulfhydryl groups) acting as common reaction sites within biological systems, sulfur-containing QDs can stably bind with biomolecules [122].

Khaliq et al. suggested that Au/CdS QDs/TNTs have the potential to be used in the non-enzymatic electrochemical detection of cholesterol and H_2_O_2_. In this study, a thin coating of CdS QD-coated titanium dioxide nanotubes (TNTs) was coated with a thin layer of gold NPs to create biosensors. This biosensor showed excellent thermal stability, enhanced shelf life, and repeatability. Additionally, blood samples were used to test the clinical performance of the biosensor for cholesterol and H_2_O_2_, which revealed maximum relative standard deviations of 1.8 and 2.3%, respectively [123].

Omar et al. developed a CdS QDs-based optical sensor by using a thin layer of amine-functionalized graphene oxide (CdS-NH_2_GO). To sense the dengue virus (DENV) E-protein, they covalently coupled a particular monoclonal antibody (IgM) to CdS-NH_2_GO. The Au/CdS-NH_2_GO/EDC-NHS/IgM film shows significant binding to DENV E-protein and great potential sensitivity [126].

Table 5 displays recent studies about the applications of CdS NPs in biosensors. In general, using these NPs can improve the properties of biosensors, including increased sensitivity, greater stability, and quick and convenient operations.

## 5. Conclusions and Future Perspectives

According to extensive studies on CdS NPs, in vitro studies sufficiently confirmed and demonstrated its mentioned biomedical properties despite a lack of adequate in vivo studies and clinical trials. The toxicity of CdS NPs for rat neurons and Purkinje cells could explain the lack of in vivo studies for the mentioned applications [107]. On the other hand, the in vitro toxicity of CdS NPs was observed in normal kidney cells. It was also discovered that the cytotoxic effects are influenced by particle size and solubility. Moreover, it appears that modifying and combining CdS NPs with biocompatible compounds can reduce the toxicity of CdS NPs in normal cells. Because the in vitro toxicity test shows more severe cell damage than the in vivo research, it appears that more research in this field is required to overcome these differences of opinion [25,134]. This lack of in vivo studies might open a new route for researchers to overcome limitations such as the toxicity of CdS NPs under in vivo conditions. Furthermore, a potential future direction for research is to conduct molecular studies aimed at identifying the specific mechanisms that activate or inhibit signaling pathways involved in the antibacterial and anticancer activity of CdS NPs. Furthermore, considering the potential of CdS NPs in biosensing, it is recommended to focus on developing biosensors based on these NPs that offer enhanced sensitivity and selectivity for detecting different biomolecules. As demonstrated, the synergistic effect of CdS NPs in combination with other compounds, such as other types of NPs, exhibited promising properties in the aforementioned fields, and it is recommended to conduct further research in this regard. Compared to the chemical and physical NPs, biogenic synthesis produced NPs that were much lower in toxicity [15]. However, the physicochemical properties of chemical and physical NPs are undeniable. According to all the issues, the green method was better than other synthesis methods because it could have similar and even more useful biomedical applications than other synthesized CdS NPs by maintaining the appropriate and sufficient structural characteristics. The reported CdS NPs were also well characterized in terms of optical, photograph, etc., characteristics with the employment of various techniques, so it can be said that CdS NPs have one of the most comprehensive characteristics among the synthesized NPs. In terms of size, a useful feature in medical applications discussed in this article, biogenic, chemical, and physical NPs were in the range of ~2–45, ~5–90, and ~5–180 nm, respectively. Moreover, structures with sizes greater than 100 nm were constructed and reported. In terms of morphology, most of the NPs were spherical, which affected their applications. CdS NPs demonstrated promising potential for biosensor, antimicrobial, bioimaging, and antibacterial applications, most of which were related to green CdS NPs. Finally, it can be concluded that CdS NPs can be considered one of the best metal NPs with various applications in the field of medicine and pharmaceutics.

## Figures and Tables

**Table 1:** A list of different methods for the synthesis of cadmium sulfide nanoparticles (CdS NPs) and characterization techniques.

	Methods	Importance of Method	Characterizations ^*^	Morphology/Size (nm)	Microorganism or Plant	Year	Ref.
Green	Green or biogenic synthesis	-Biocompatible; -Eco-friendly; -Quick process; -Economically affordable; -One-pot synthesis; Least hazardous; Does not require the use of stabilizing agents.	UV–visible spectra, SEM, EDX, FTIR, XRD	-	*Escherichia coli*	2020	[23]
			UV–visible spectra, FESEM, XRD, FTIR	Spherical/~19.07±2.54	*Lactobacillus acidophilus*	2022	[27]
			UV–visible spectra, XRD, FTIR, TEM	non-spherical/5 ± 0.4 (Protein-capped NPs) and 11 ± 0.75 (Bare NPs)	*Escherichia coli*	2015	[28]
			UV–visible spectra, SEM, EDX, TEM, XRD	Spherical/6–15	*Trichosporon jirovecii*	2015	[29]
			TEM, XRD, XPS	Spherical/4–12	*Viridi bacillus arenosi* K64	2021	[30]
			UV–Vis spectra, TEM, EDX, SEM	-/15–20	*Escherichia coli*	2017	[31]
			UV-Vis and fluorescence spectra, TEM	Spherical/7–15	*Fusarium oxysporum f.* sp. *lycopersic*	2017	[32]
			UV-Vis spectra, HRTEM, EDS, FTIR	Spherical/3–6	*Rhodopseudomonas palustris* TN110	2019	[33]
			UV–visible spectra, SEM, EDX, FTIR, XRD	-	*Bacillus licheniformis*	2015	[34]
			XRD, FTIR, TEM, FESEM, EDX	Spherical/~15	*Shewanella oneidensis*	2017	[35]
			UV-Vis spectra, EDX, FTIR, XRD, DLS, TEM, Fluorescence spectrophotometer	Spherical/2–10	*Aspergillus niger*	2020	[36]
			TEM, SEM, EDX	-/-	*Pseudoalteromonas* sp. MT33b	2021	[37]
			UV-Vis spectra, TEM, EDX, FTIR	Spherical/3.2–44.9 (*E.coli*) and 5.7–26.3 (*K. pneumonia*)	*Escherichia coli* E-30 and *Klebsiella pneumoniae* K-6	2018	[38]
			AFM, UV–visible spectra	-/The average size by *P. aeruginos, B. lichenisformis, E. coli, F. oxysporum & A. terrus,* was 17.86, 17.00, 17.86, 18.73 and 13.21 nm respectively	*Pseudomonas aeruginosa*, *Bacillus* *Lichenisformis*, *Escherichia coli*, *Fusarium oxysporum* and *Aspergillus terrus*	2016	[39]
			SEM	-/15–20	*Bacillus licheniformis*	2019	[40]
			UV–visible spectra, FTIR, XRD, EDX, SEM, TEM	Spherical/2.5–8	*Dicliptera Roxburghiana* plant	2021	[41]
			UV–Vis spectra, FTIR, XRD, SEM, XRF, TGA	-/~4.6 estimated by XRRD pattern	*Panicumsarmentosum*	2019	[42]
			SEM, EDX, HRTEM, FTIR, UV-Vis, and fluorescence emission spectra	Spherical/2–5	*Camellia sinensis*	2018	[43]
Chemical	Chemical precipitation method	-Simple; -Short reaction time; -Requires a hazardous chemical agent; -Able to control the size and stability of NPs.	UV–visible spectra, XRD, FTIR, TEM, SEM, EDS, TGA, DTA, DTG	Spherical/<10	-	2015	[44]
			UV–visible spectra, XRD, FESEM, FTIR, PL, Raman measurement	Spherical/ few hundred to tens of nanometres	-	2015	[45]
			XRD, FESEM, HRTEM, UV–visible spectra, Micro-Raman measurements, EDS	Spherical/15–85	-	2015	[46]
			UV–visible spectra, XRD, SEM, FTIR, EDX	Nano-rods/14.3–18.7 Spherical/6–8.8	-	2018	[47]
			UV-visible spectra, Raman Spectra, XRD, FESEM, EDX	Spherical/6.8–28	-	2020	[48]
			XRD, SEM, TEM, UV–visible spectra, PL, DLS	Spherical/15–42	-	2016	[49]
	Wet chemical synthesis	-Simple; One step; -Does not require a high temperature and potential chemical agent; -Quick process.	TEM, ICP-AES	-/5–10	-	2019	[50]
			UV–visible spectra, XRD, FTIR, TEM, XPS, PL, DLS	Spherical/10-15	-	2019	[25]
			UV-Vis spectra, TEM, EDX, FTIR	-/8.77–16.50	-	2018	[38]
	Solvothermal synthesis	-Control of morphology, dimensions, and structure of nanomaterials; -Multiple steps; -Fabricates pure and clean nanoparticles with a high degree of crystallinity	XRD, XPS, FETEM, FESEM, UV/VIS/NIR spectra, PL	Irregular particle shape/Small particles less than 5 nm and large particles greater than 10 nm	-	2017	[51]
	Chemical reduction method	-Inexpensive; -Simple;	UV-Vis-NIR spectra, XRD, FTIR	-/-	-	2017	[52]
	-Formation of various morphologies; -Requires a high temperature; -Synthesis of the relatively stable particles that can be re-dispersed in nonpolar solvents easily.	-Formation of various morphologies; -Requires a high temperature; -Synthesis of the relatively stable particles that can be re-dispersed in nonpolar solvents easily.	XRD, FESEM, TEM, EDX, PL, CHNS elemental analyzer, TG/DTA, DRS	-Thermal decomposition of cadmium thiourea complexes in diphenyl ether: microspheres, pyramid-like and mixture of nanorods and NPs. -Solid state thermal decomposition of different cadmium thiourea complexes: microspheres, nanotube-like, flower-like and irregular shape	-	2015	[53]
			XRD, FTIR, TGA, EDX, FESEM, TEM, DRS, PL, UV–Vis–NIR spectra	-Pyramid-like morphology/ The height of the pyramid is about 400 nm, and the diameter of the base is about 300 nm -Sponge-like morphology/200–300 nm -Hexagonal disc-like particles/50–70 nm -Flower-like nanostructures/250–300 nm -Gypsum rose and rosette-like particles/300-400 nm	-	2015	[54]
			XRD, SEM, TEM, FTIR, PL	-/30–40	-	2016	[55]
	Sol–gel method	-An appropriate method for the development of quality crystals with high surface area and different morphologies; -Fast and simple.	XRD, PL, SEM, HRTEM, FTIR	Spherical/24	-	2020	[56]
			XRD, FESEM, TEM, EDS, FTIR, PL	Spherical/<10	-	2020	[57]
			XRD, TEM, UV–visible spectra	Spherical/9 (pure CdS) and 16 (Ni-doped CdS)	-	2018	[58]
	Combustion	-Short-time reaction; -Does not require high temperature.	XRD, TEM, FTIR, PL, BET, DRS	-/6 nm for rinsed and 3 nm for washed samples	-	2015	[59]
	Sono-chemical Method	-Rapid reaction rates; -Controllable reaction condi-tions; -Able to form nanoparti-cles with high purity; -Quick in process.	XRD, TEM, EDX	Spherical/10	-	2016	[60]
			XRD, TEM, EDX	Hexagonal platelets/19.3–22.9	-	2015	[61]
			UV–visible spectra, FTIR, XRD, TGA, DLS, SEM, TEM	-/6	-	2015	[62]
	Micro-emulsion method	-Multiple steps; -Does not require certain conditions.	TEM, UV-visible spectra	Spherical/49–89	-	2013	[63]
Physical	Pulsed laser ablation	-Facile; -Ecofriendly; -Able to control size and shape.	XRD, TEM, XPS, PL, UV–visible spectra	Spherical/21 ± 9.1	-	2016	[64]
			UV–visible spectra, XRD, SEM, AFM	Spherical, monopod, bipod, and tripod rods/40 nm for NPs synthesized with 1.76 J/cm^2^ (2.8–5.5) µm in length and (80–400) nm in diameter for NPS synthesized with 2.25 J/cm^2^	-	2015	[65]
			XRD, TEM	-/10–15	-	2015	[66]
	Hydrothermal method	-Highly pure, with controlled morphology of NPs; -Has a narrow size distribution and consists of single crystals; -Production of fine-grained powder; -High reaction rate of powders; -Good dispersion in liquid; -Almost pollution-free; -Does not require expensive and highly sophisticated equipment;	XRD, FESEM, EDS, FTIR, UV–Diffuse Reflectance spectroscopy, PL	Spherical/ 50.8	-	2018	[67]
			UV–visible spectra, XRD, SEM	Spherical/50 & 150	-	2022	[68]
			XRD, SEM, XPS, UV–vis diffuse reflectance spectroscopy, PL, BET	-/~25	-	2019	[69]
	Microwave-assisted	-Short reaction time; -Gives a narrow particle size distribution of nanocrystals with a high purity; -Cheap; -Environmentally friendly.	XRD, UV–visible spectra, SEM, TEM	-/8–10	-	2016	[70]
			SEM, XRD, FTIR	-/75–180 (uncapped CdS) 40–59 (PVP-capped CdS)	-	2016	[71]
			XRD, FESEM, TEM, EDX, PL	Spherical/15–25	-	2019	[72]
			XRD, TEM, PL, UV–visible spectra	-/30–60	-	2018	[73]
			XRD, TEM, UV-Vis spectra	Spherical/8.9 nm at 10 min and 9.2 nm at 15 min irradiation time.	-	2013	[74]
	Reflux method	-Simple; -Low cost; -Aqueous based.	XRD, TEM, FTIR, STEM, XPS, PL	-/5–8	-	2020	[75]
Physico-chemical	Mechanochemical processes	-The reaction occurs at low temperatures; -Particle size can be controlled by changing milling conditions and starting materials; -Reproducible; -Ensures a high yield -Simple and easy to operate.	XRD, SEM, UV/Vis/NIR Spectra, EDX, BET	-/-	-	2018	[76]
			XRD, HRTEM	- / ~5	-	2016	[77]
			XRD, XPS, PL, UV–visible spectra, TEM, EDS, DLS, Raman spectroscopy	-/<10	-	2022	[78]

* UV–Vis spectra, ultraviolet–visible spectrophotometry; SEM, scanning electron microscopy; EDX/EDS/EDAX, energy-dispersive X-ray spectroscopy; FTIR, Fourier-transform infrared spectroscopy; XRD, X-ray diffraction; TEM, transmission electron microscopy; XPS, X-ray photoelectron spectroscopy; HRTEM, high-resolution transmission electron microscopy; FESEM, field-emission scanning electron microscopy; AFM, atomic force microscopy; TGA, thermal gravimetric analysis; PL, photoluminescence; UV/Vis/NIR spectra, ultraviolet/visible/near-infrared spectroscopy; DLS, dynamic light scattering, DTA: Differential Thermal Analysis, DTG: derivative thermogravimetry, ICP-AES: Industry coupled plasma emission spectrometry, DRS: diffuse reflectance spectroscopy, BET: Brunauer–Emmett–Teller.

**Table 2:** The anticancer applications of CdS NPs.

Type of CdS NPs	Synthesis Method	Morphology and Size (nm)	Cancer (Cell Line)	Effects	Explanations	Year	Ref.
CdS QDs	Green synthesis (*Camellia sinensis*)	Spherical/2–5	Human lung alveolar basal epithelial cell line (A549)	- With CdS QDs, the inhibition of A549 cells is gradually enhanced (A549 cell viability at 50 g/mL was 20%), and the effect is comparable to that of the medication cisplatin (A549 cell viability at 50 g/mL was 24%).	Owing to the high florescence emission and quantum confinement effect results, green-synthesized CdS QDs particles could interact with the phosphorous moieties in DNA, and then DNA replication is inactivated.	2018	[43]
CdS NPs	Green synthesis (*Shewanellaoneidensis*)	Spherical/15	Rat glioma cell line (RG2)	- The cytotoxic effect of the biosynthesized CdS NPs in the presence of IL increased with increasing NP concentrations. (Cell viability of GR2 in 100 μM CdS NPs was 75% and for CdS/IL NPs was 65%).	The improved cellular uptake was due to the improved surface morphology and surface area of the NPs via the IL soft template action.	2018	[35]
Gallic acid/cadmium sulfide (GA/CdS) NPs fabricated on graphene oxide (GA/CdS-rGO) nanosheets	-	Spherical CdS NPs	Human glomerular mesangial cancer cells (IP15)	- In samples treated with GA/CdS-rGO, the number of viable cells was decreased, and 83.87% inhibition was seen. - The IC50 value for IP15 cells was 50 µg/mL, and 55.05% of inhibition was obtained in pure CdS nanoparticles on IP15 cells.	- Oxidative stress from ROS species, mitochondrial dysfunction, and an increase in intracellular Ca^2+^ levels are associated with the apoptosis of cancer cell types caused by CdS/GA. - The anticancer properties of GA/CdS nanocomposites are superior to those of unprocessed CdS NPs.	2018	[109]
CdS/rGO NPs (graphene oxide/CdS nanocomposite)	Solvothermal method	Spherical CdS NPs /~10	Hela cells	- The IC50 value of the CdS NPs on the normal and cancer cells is about around 60 μg/mL.	- The characteristics of the nanocomposites are improved by adding CdS to the rGO matrix. - The created CdS/rGO nanocomposites were likewise very effective at killing tumor cells.	2019	[110]
ZnO-CdS NPs	Chemical synthesis	-/-	- Hepatocellular carcinoma (HepG2) - Mammary gland (MCF-7) - Epidermoid carcinoma (HEP2) - Colorectal carcinoma (HCT-116) - Rhabdomyosarcoma (RD)	- IC50 of ZnO-CdS NPs against human tumor cells HePG2, HCT-116, MCF-7, RD, and HeP2 was 9.26 µg, 5.64 µg, 7.90 µg, 9.51 µg, 10.17 µg, respectively.	- The anticancer results of ZnO-CdS NPs were comparable to the anticancer results of doxorubicin. - Probably the pathway of treatment with ZnO/CdS nanocomposites was based on ROS production.	2019	[111]
composite of Cd loaded on ZnO	Pulsed laser ablation in water media	Spherical/12	Human colorectal carcinoma cells (HCT-116)	- The IC50 values for 10% CdS/ZnO, (0.10 µg/mL), 20% CdS/ZnO, (0.12 µg/mL), and CdS (O.13 µg/mL).	- CdS-loaded ZnO showed better anticancer activities than CdS.	2020	[112]
CdS NPs	Green synthesis (*Aspergillusniger*)	Spherical/2–10	- Breast cancer (MCF7) - Lung cancer (A549) - Prostatic carcinoma (PC3)	- 50% inhibitory concentrations (IC50) CdS NPs against MCF7, PC3, and A549 cell lines of 190 g/mL, 246 g/mL, and 149 g/mL, respectively.	- Long-term exposure of CSNPs to an oxidizing environment can lead to CSNP decomposition and the release of Cd ions.	2020	[36]
CdS NPs	Green synthesis	-/-	*Mus musculus* skin melanoma (B16F10) and Human epidermoid carcinoma (A431)	- The cytotoxicity of CdS NPs on A431 cells was inhibited by 81.53% at 100 M, which was significantly more effective than 5-ALA, which inhibited A431 cells by 33.45% at 1 mM.	- CdS NPs have a less toxic effect on musculus skin melanoma (B16F10) than epidermoid carcinoma (A431) cell lines. - CdS NPs showed more cytotoxic effects on cancer cells compared with standard 5-aminolevulinic acid (5-ALA).	2020	[23]

**Table 3:** The antimicrobial applications of CdS NPs.

Type of CdS NPs	Synthesis Method	Morphology/Size (nm)	Microorganism	Test Approach	Results	Year	Ref.
CdS NPs	Green synthesis (*Pseudomonas pseudoalcaligenes* strain Cd11)	Spherical/12–19	- *Escherichia coli* - *Bacillus subtilis* - *Staphylococcus aureus* - *Pseudomonas aeruginosa* - *Lactobacillus plantarum* - *Pseudomonas fluorescens*	Well-diffusion method	- The inhibitory effect of CdS NPs was observed. - The highest inhibitory effect on *L. Plantarum* was 2 mm inhibition The lowest inhibition was with inhibition and for *P. Fluorescens*. - The inhibitory effect on other bacteria studied was between 1 and 2 mm.	2018	[115]
CdS NPs	Green synthesis (*Escherichia coli* and *Klebsiella pneumoniae*)	Spherical/3.2–44.9 (*E.coli*) and 5.7–26.3 (*K. pneumonia*)	- *Aspergillus fumigatus* - *Aspergillus niger* - *Geotricum candidum* - *Candida albicans* - *Bacillus subtilis* - *Streptococcus pneumoniae* -*Staphylococcus aureus* -*Staphylococcus epidermidis* - *Pseudomonas aeruginosa* - *Escherichia coli* - *Proteus mirabilis* - *Klebsiella pneumoniae*	Well-diffusion method	- CdS NPs synthesized had the maximum zone of inhibition against *Bacillus subtilis* (23.4 mm), *Staphylococcus aureus* (22.1 mm), *Pseudomonas aeruginosa* (21.4 mm), *Escherichia coli* (17.3 mm), *Geotricum candidum* (17.3 mm), *Aspergillu sfumigatus* (17.3 mm), and *Aspergillus niger* (14.7 mm). -In comparison to chemically produced CdS NPs, biogenic CdS NPs exhibited the highest levels of inhibition on the majority of strains. - Gram-positive bacteria displayed the strongest inhibition, followed by Gram-negative bacteria.	2018	[38]
CdS NPs	Green method (*Panicum sarmentosum*)	-/~4.6 estimated by XRD pattern	-*Staphylococcus aureus* -*Escherichia coli*	Well-diffusion method	- CdS NPs showed antibacterial efficacy against *Staphylococcus aureus* and *Escherichia coli*. - The antibacterial property and the diameter of the bacterial inhibition zone increased with the increased dosage of the NPs. Gram-negative bacteria (*Escherichia coli*) were discovered to have higher CdS NPs resistance than Gram-positive bacteria (*Staphylococcus aureus*).	2019	[42]
Cobalt doped CdS NPs	Chemical method	Spherical/15–20	*-Escherichia coli* - *Staphylococcus aureus*	Disk-diffusion method	- While Gram-positive bacteria can withstand the antibiotic effects of CdS nanoparticles, Gram-negative bacteria are killed by them. - The diameter of the zone for Gram-positive bacteria (30–40 mm). - The diameter of the zone for Gram-negative bacteria (6–41 mm).	2020	[116]
CdS NPs	Green synthesis (*Aspergillus niger*)	-/-	- *Escherichia coli* - *Bacillus licheniformis* - *Pseudomonas aeruginosa* - *Bacillus cereus* - *Staphylococcus aureus* - *Fusarium oxysporum* - *Aspergillus flavus* - *Penicillium expansum*	Well-diffusion methods	- *Bacillus licheniformis, Escherichia coli, Bacillus cereus,* and *Pseudomonas aeruginosa* have inhibition zones of 25.1 mm, 23.5 mm, 20.6 mm, and 13.6 mm, respectively. - CdS NPs outperformed common antibiotics ampicillin, trimethoprim, and cefotaxime in their ability to inhibit *Pseudomonas aeruginosa, Bacillus cereus,* and *Escherichia coli*. - Inhibition zone *on Penicillium expansum* 18.6 mm, *Fusarium oxysporum* 23.0 mm, and *Aspergillus flavus* 29.7 mm. - CdS NPs showed greater activity than fluconazole in *Penicillium expansum*.	2020	[23]
CdS NPs	Green synthesis (*Aspergillus niger*)	Spherical/2–10	- *Escherichia coli* - *Pseudomonas vulgaris* - *Staphylococcus aureus* - *Bacillus subtilis* - *Candida albicans*	Well-diffusion method	- Gram-positive bacteria were more affected by CdS NPs than Gram-negative ones, and CdS NPs had a substantial antibacterial effect on all of the bacterial pathogens that were studied. - Antimicrobial activity inhibition zone for *Bacillus subtilis* was 16 mm; for *Staphylococcus aureus*, it was 25 mm; for *Escherichia coli*, it was 14 mm; and for *Proteus vulgaris*, it was 16 mm. -No evidence of antimicrobial activity against *Candida albicans* was found.	2020	[36]
CdS QDs	Chemical and Green methods (synthesized by *Fusarium oxysporum* f. sp. *lycopersici*)	Spherical/4.08 ± 0.07 nm for biogenic NPs and 3.2±0.20 nm for chemical NPs	*E. coli*	Well-diffusion method	- In bacterial cells, biogenic CdS QDs had a less lethal effect than chemical CdS QDs. - As the NPs’ concentration increased, a decrease in cell viability was seen. - Compared to the control and the biological NPs, minimal cellular viability was obtained for the chemical nanoparticles in all treatments.	2021	[117]

**Table 4:** The bioimaging applications of CdS NPs.

Type of CdS NPs	The Synthesis Method of CdS NPs	Size (nm)	Type of Microscope/Cell type	Color	Results	Year	Ref.
- Halloysite nanotubes (HNTs): - HNTs-Azine-CdS - HNTs-NH2-CdS - HNTs-Azine-Cd0.7Zn0.3S	Cadmium sulfide and cadmium–zinc-sulfide QDs were stabilized on the halloysite.	6–8	Laser scanning microscopy/PC-3 cells	- Green (HNTs-Azine-CdS) - Yellow-red (HNTs-Azine-Cd_0.7_Zn_0.3_S) - Red (HNT-NH_2_-CdS)	- Bright and well-resolved fluorescence was observed in all cases. - Well distributed on the cells’ surfaces or inside them.	2018	[120]
			Dark-field and epifluorescence microscopy/PC-3 cells	NT-NH_2_-CdS bright white spots, or yellow and red spots	- Appear as bright spots due to their good light-scattering properties. - The cell membrane and cytoplasm can also be seen.		
CdS QDs	Green synthesis using tea leaf extract (*Camellia sinensis*)	2–5	Fluorescence microscopy/A549 cancer cells	Yellow, red, and orange fluorescence correspond to early apoptotic cells, necrotic cells, and late apoptotic cells, respectively.	- CdS QDs produce effective intracellular fluorescence intensity. - The fluorescence emission is enhanced with CdS QDs concentration. This enhanced fluorescence is responsible for the high-contrast fluorescence bioimaging.	2018	[43]
CdS NPs and Chitosan-coated CdS NPs	Wet chemical method	10–15	Fluorescence microscopy/Jurkat cells	-	- Cell images are not clear enough; this might be due to the low incorporation of Chitosan-coated CdS NPs.	2019	[25]
CdS QDs capped with dextrin and bioconjugated with doxorubicin	Chemical synthesis	5	Confocal laser fluorescent microscope/HeLa cells	Green and red spectrum	- In cells treated with CdS-Dx/DOX QDs, the fluorescence was observed mainly in the cytoplasm and in lesser amounts in the nucleus. - The changes in the morphology of those cells treated with DOX alone and CdS-Dx/DOX QDs were clearly evident. - The high photostability of CdS-Dx/QDs, both alone and conjugated.	2020	[105]
CdS NPs	Green synthesis (*Chromolaenaodorata, Plectranthusamboinicus*, and *Ocimumtenuiflorum*)	-	Cell imaging with bright-field and fluorescence microscope/HeLa cells	Hela cells showed bright green fluorescence	- The use of cadmium sulfide nanoparticles synthesized by the green method is more suitable due to the lower toxicity of these compounds for cells.	2021	[119]
CdSAg NPs	Green synthesis (*E. coli*)	5.49 nm for CdS NPs and 7.20 nm for CdSAg NPs	Confocal microscope/HeLa cells	Red fluorescent	- It is not associated with changes in the cell morphology. - The fluorescence is stable.	2021	[121]

**Table 5:** The biosensor applications of CdS NPs.

Type of CdS NPs	Synthesis	Basis of the Test	Measured Substance	Detection Limit	Benefits	Year	Ref.
CdS QDs	CdS/WS_2_ nanosheets modified ITO electrode surface	Photoelectrochemical	DNA	5 fM to 50 pM	This biosensor showed excellent analytical performances under optimized conditions, low detection limit, favorable selectivity, and satisfactory stability.	2019	[127]
CdS QDs	CdS QDs capped with Chitosan and Bioconjugated with enzyme	Electrochemical	Cholesterol	0.64–12.9 mM	The synthesis of “enzyme-QDs-polymer” system is platform in bionanocomposite formation for electrochemical applications.	2015	[128]
CdS QDs	The CuO inverse opal photonic crystals were synthesized by the sol–gel method and modified with CdS QDs by successive ionic layer adsorption and reaction (SILAR).	Photoelectrochemical	Glucose	up to 4345μA mM^−1^ cm^−2^	It showed strong stability, good reproducibility, excellent selectivity, and fast amperometric response.	2015	[129]
CdS QDs	The chiral CdS QDs (DPA/Cys-CdS QDs) were prepared by mixing cysteamine-capped CdS QDs (Cys-CdS QDs; achiral QDs) with D-penicillamine (DPA).	Circular dichroism spectroscopy (CD)	Glucose	50–250 μM	- Detection of glucose by indirect measurement of the concentration of H2O2 generated by the enzymatic reaction of GOx and glucose.	2018	[124]
CdS QD	Core–shell CdTe/CdS QDs were synthesized by a simple one-pot chemical reduction method	Fluorescence resonance energy transfer (FRET)	Mercury	0.1 nM to 2 μM	CdTe/CdS QDs exhibit fluorescence quenching as the mercury concentration increases, acting as an “OFF-sensor.”	2018	[130]
CdS nanocrystals	CdS-Au NPs were made by three different methods, namely the quenching method (QM), amplification method (AM), and ratiometric method (RM).	Electrochemiluminescence	Thrombin	QM:>92 pg.mL^−1^	RM showed great selectivity and good authenticity in real samples.	2019	[131]
				AM: >6.5 pg.mL^−1^			
				RM: >500 fg.mL^−1^			
CdS QDs	Au/CdS-NH_2_GO/EDC-NHS/IgM thin film was prepared and deployed in an SPR-based optical sensor	Surface plasmon resonance (SPR)	Dengue virus E-protein	0.001 nM/1 pM	- CdS-NH_2_GO thin films are beneficial to the improvement of the performance of SPR biosensor.	2019	[126]
CdS NPs	Modified electrode containing cadmium sulfide CdS NPs (CdS@enrofloxacin-tetraphenylboron)	Electrochemical	Enrofloxacin (ENR)	10^−2^–10^−7^ mol·L^−1^	- Good selectivity. - Reproducibility. - Response time (<40 s). - Lifetime (up to 12 weeks). - A pH range (3.3–7.2). - Can be a reference for ENR rapid and efficient determination.	2019	[125]
CdS QDs	A thin layer of Au NPs sputtered on CdS-QDs-decorated anodic titanium dioxide nanotubes (TNTs) was fabricated (Au/CdS QDs/TNTs)	Electrochemical	Cholesterol	0.024−1.2 mM	- Good reproducibility. - Thermal stability. - Increased shelf life.	2021	[123]
			H_2_O_2_	18.73−355.87 μM			
CdS QDs	Potato extract was used as a stabilizer and modifier to synthesize CdS QDs (green synthesis)	Fluorescence resonance energy transfer (FRET)	Ag^+^	1–100 mg/L	For the prepared CdS QDs, a good fluorescence quenching effect was observed, indicating its potential application for the rapid detection of Ag^+^.	2021	[132]
CdS nanoarrays	Heterogeneous cuprous-oxide-coated silver (Ag@Cu_2_O) nanocomposites/graphitic carbon nitride (g-C3N4)/CdS nanoarrays structure was constructed	Photoelectrochemical (PEC)	Carcinoembryonic antigen (CEA)	10^−5^–1 ng/mL	- High sensitivity. - Excellent anti-interference ability. - Favorable repeatability. - Good stability.	2022	[133]

## Data Availability

No new data were created.
